# Microwave Tomography Using Neural Networks for Its Application in an Industrial Microwave Drying System

**DOI:** 10.3390/s21206919

**Published:** 2021-10-19

**Authors:** Rahul Yadav, Adel Omrani, Guido Link, Marko Vauhkonen, Timo Lähivaara

**Affiliations:** 1 Department of Applied Physics, University of Eastern Finland, FI-70210 Kuopio, Finland; marko.vauhkonen@uef.fi (M.V.); timo.lahivaara@uef.fi (T.L.); 2 Institute for Pulsed Power and Microwave Technology, Karlsruhe Institute of Technology (KIT), 76133 Karlsruhe, Germany; adel.hamzekalaei@kit.edu (A.O.); guido.link@kit.edu (G.L.)

**Keywords:** microwave drying, moisture content distribution, microwave tomography, inverse problems, neural networks

## Abstract

The article presents an application of microwave tomography (MWT) in an industrial drying system to develop tomographic-based process control. The imaging modality is applied to estimate moisture distribution in a polymer foam undergoing drying process. Our Leading challenges are fast data acquisition from the MWT sensors and real-time image reconstruction of the process. Thus, a limited number of sensors are chosen for the MWT and are placed only on top of the polymer foam to enable fast data acquisition. For real-time estimation, we present a neural network-based reconstruction scheme to estimate moisture distribution in a polymer foam. Training data for the neural network is generated using a physics-based electromagnetic scattering model and a parametric model for moisture sample generation. Numerical data for different moisture scenarios are considered to validate and test the performance of the network. Further, the trained network performance is evaluated with data from our developed prototype of the MWT sensor array. The experimental results show that the network has good accuracy and generalization capabilities.

## 1. Introduction

**Figure 1:**
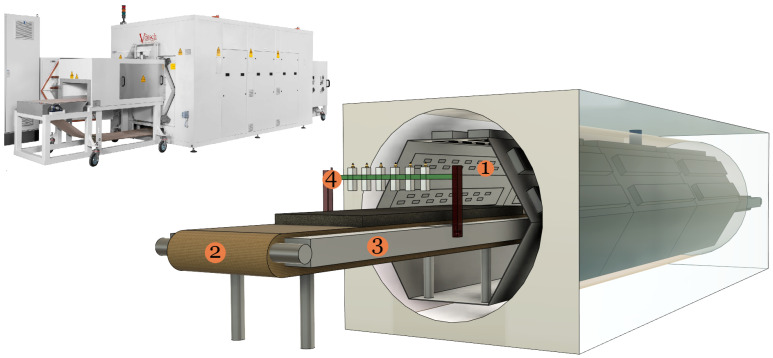
**Left**: view of the HEPHAISTOS microwave oven system. **Right**: schematic showing the main modules of the oven which are represented by numbers tag 1, 2, 3, and 4. Tag 1 is high power microwave waveguide antenna, Tag 2 is the conveyor belt, and Tag 3 is the metal plate. MWT setup with waveguide antenna is represented by Tag 4. The foam is shown as dark gray matter and placed on the conveyor belt.

Controlled/localised heating in industrial microwave oven [1,2] is paramount to address hot-spot formation and thermal runaway issues [3]. As a consequence, system efficiency and processed product quality may improve. Presently, we are working on a type of microwave oven technology called HEPHAISTOS, as shown in Figure 1. The system is characterized by hexagonal geometry [4] for the cavity that supports a very high electromagnetic field homogeneity. Its principal areas of applications are in material processing such as thermal curing of fiber composites and drying of porous foams. Specifically, during drying of a porous polymer foam, thermal runaway and hot-spot formation may occur [5,6]. Such situations may lead to low-quality processing and may even damage the industrial unit in case a fire is kindled in the foam. Therefore, automatic online control of power sources (magnetrons) to obtain a selective heating rate at each stage of the drying process is one option to eliminate these problems. To apply such precise control of power sources, non-invasive in situ measurement of the unknown distribution of moisture, especially dominant wet-spots, inside the material is required. The infrared temperature sensors integrated with the microwave drying systems are capable of giving information only on the surface of the material. That is not sufficient to provide efficient control of microwave sources. Thus, integration of microwave tomography (MWT) imaging modality operating in X-band range [7] (from 8 GHz to 12 GHz) with the drying system is proposed (see number Tag 4 in Figure 1) to estimate the moisture content distribution in a polymer foam. Based on the MWT tomographic output, an intelligent control strategy for power sources can be derived. Preliminary work in this direction is reported in [8] by the authors. Industrial process tomography based on microwave sensors for various applications are reported in [9,10]. The specific use-case of microwave sensor technology for moisture measurements in a sample are given in [11,12,13,14,15,16,17]; but they are limited in providing moisture information on the surface or in a small sample size but not the volumetric information as required for our purpose.

For MWT, real-time image reconstruction is critical to provide a fast input response for the control system. In addition, the inverse scattering problem that we are solving is severely ill-posed due to the large object size and inhomogeneous profile. Thus, applying iterative optimization-based methods like Levenberg–Marquardt [18], contrast source inversion, and subspace-based optimization methods [19] are time-consuming. An attractive approach to fulfill the real-time estimation requirement is to use neural networks [20,21]. The first implementation of neural networks, to the best of our knowledge, in solving an inverse problem in electromagnetics where material properties of multilayered media is estimated was presented in [22]. In [23,24], artificial neural network is employed for determining the moisture content in wheat and moisture content of commercially important biomass, respectively.

Recent developments in the use of neural networks for solving general microwave imaging problem are detailed in [25,26,27,28,29,30]. In [31], a connection between the optimization framework and neural network is established and tested to solve nonlinear inverse scattering problems. However, they are limited to sparse target recovery with full-angle sensor configuration and a large number of measurements. In this work, an MWT configuration with antennas located only on top is chosen as a setup to support fast data acquisition. Secondly, our network is trained using the smoothness parameter model to represent possible moisture distribution scenarios and is capable of even generalizing sparse targets as shall be demonstrated by the experimental results. Using ideas from our preliminary studies [32,33,34,35], we build a comprehensive synthetic dataset consisting of different moisture content distribution scenarios and the corresponding scattered electric field responses using two-dimensional (2-D) method-of-moment formulation. Once the selected network architecture is trained using this dataset, it is applied to recover the moisture content distribution (in terms of dielectric constant) in real-time. The performance of trained network is validated with the numerical MWT data for different moisture scenarios. Further, the network is tested on the experimental data from the MWT setup integrated with the drying unit. Results presented shows that neural network approach can successfully estimate the moisture content in real-time.

The paper is organized as follows: The forward model for MWT problem and its formulation is detailed in Section 2. Furthermore, Section 2 also details the parametric model for generation of moisture distribution. Section 3 details the neural network based approach in the MWT and numerical results are presented. The experimental setup of the MWT is detailed in Section 4 and performance of the neural network with the experimental data is tested. Discussion and concluding remarks are given in Section 5.

## 2. Problem Formulation

To generate the numerical dataset for the neural network, we begin our discussion by first unveiling the scattering model of the problem. With reference to the MWT measurement schematic shown in Figure 1, we chose to illustrate the scattering model in the context of its 2-D configuration. The 2-D configuration is chosen instead of 3-D model as to decrease the overall computational load for generating the dataset.

### 2.1. Forward Model

**Figure 2:**
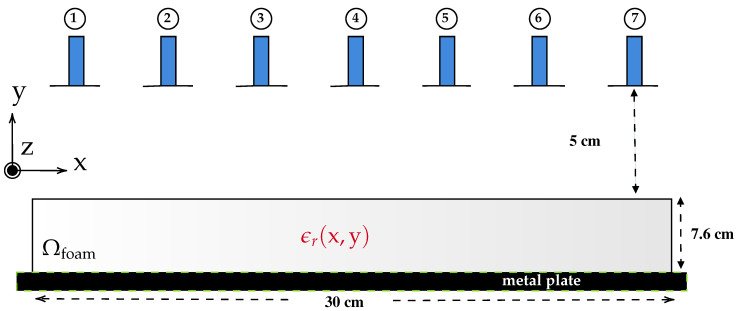
2-D schematic of the MWT setup with waveguide antennas denoted by number from 1, 2, *…* 7.

The 2-D cross-section of the MWT setup is shown in Figure 2 with multistatic measurement configuration. In the figure, we consider a two-dimensional foam domain $\Omega_{\mathrm{foam}}=\left[-15,\;15\right]\times \left[0,\;7.6\right]$ cm with inhomogenous relative dielectric constant $\epsilon_{r}=\epsilon_{r}^{\prime }-j\epsilon_{r}^{\prime \prime }$. The foam is placed on the metal plate (as shown in Tag 4 in Figure 1) which is modeled here as perfect electric conductor (PEC) plane and surrounded by background domain Ω consisting of air with $\epsilon_{r}=1-j0$. For this 2-D numerical study, the waveguide antennas are modeled as a *z*-oriented electric line source [36]; 7 such line sources are placed in a transceiver mode at a distance of 5 cm from the top surface of the foam.

In general, the scattered electric field under the illumination of time-harmonic (time convention $e^{-j\omega t}$ with angular frequency ω is used and suppressed) transverse magnetic (TM) *z*-polarized incident field is governed by the following coupled scalar volume integral Equations (VIEs) [37,38,39,40,41]

(1) $$\begin{array}{c} E_{\mathrm{sct}}\left(r\right)=k^{2}\int_{\Omega_{\mathrm{foam}}}G\left(r,r^{\prime }\right)\left(\epsilon_{r}\left(r^{\prime }\right)-1\right)E\left(r^{\prime }\right)dr^{\prime },\\ \;\forall r\in \Omega ,\;r^{\prime }\in \Omega_{\mathrm{foam}}. \end{array}$$

The term $E_{\mathrm{sct}}$ is the scattered electric field. The wavenumber of the background medium is denoted by *k*. The term $G(r,r^{\prime })$ is the free-space Green’s function. The source and the observation points are denoted by the position vectors r↦(x,y) and $r^{\prime }\mapsto \left(x^{\prime },y^{\prime }\right)$, respectively. The term *E* is the total electric field inside the scattering object and is given as

(2) $$\begin{array}{c} E\left(r\right)=E_{\mathrm{inc}}\left(r\right)+k^{2}\int_{\Omega_{\mathrm{foam}}}G\left(r,r^{\prime }\right)\left(\epsilon_{r}\left(r^{\prime }\right)-1\right)E\left(r^{\prime }\right)dr^{\prime },\\ \;\forall r,r^{\prime }\in \Omega_{\mathrm{foam}}, \end{array}$$

where $E_{\mathrm{inc}}$ is the incident electric field from the line-source. The effects of the conducting plane are included in the 2-D free-space Green’s function of the VIEs by the use of half-space Green’s function [42]. It is defined using image theory principle [43] where an image source is introduced to account for the reflections from the surface of the conducting plane and thus the conducting plane can be removed. The image source point (denoted here as $x_{\mathrm{im}}$ and $y_{\mathrm{im}}$) must have the same magnitude as the actual source, its phase must be 180 degree out of phase from the actual source and it must be placed below the conducting plane at a depth $y_{\mathrm{im}}=-y$. Such a system configuration does lead to zero tangential electric field [44] along the *x*-direction. The half-space Green’s function includes both the primary contribution $G_{T}\left(r,r^{\prime }\right)$, which is the free-space Green’s function, and the secondary contribution $G_{R}\left(r,r_{\mathrm{im}}^{\prime }\right)$ due to the image source and denoted as

(3) $$G\left(r,r^{\prime }\right)=G_{T}\left(r,r^{\prime }\right)+G_{R}\left(r_{\mathrm{im}},r^{\prime }\right).$$

Therefore, the scattered electric field above the conducting plane (i.e., upper half-space y>0) is equal to

(4) $$\begin{array}{c} E_{\mathrm{sct}}\left(r\right)=k^{2}\int_{\Omega_{\mathrm{foam}}}G_{T}\left(r,r^{\prime }\right)\left(\epsilon_{r}\left(r^{\prime }\right)-1\right)E\left(r^{\prime }\right)dr^{\prime }\\ \;+k^{2}\int_{\Omega_{\mathrm{foam}}}G_{R}\left(r_{\mathrm{im}},r^{\prime }\right)\left(\epsilon_{r}\left(r^{\prime }\right)-1\right)E\left(r^{\prime }\right)dr^{\prime }. \end{array}$$

Given the integral equation for the scattered electric field and total electric field, we resorted to discrete dipole approximation with pulse basis and point matching technique, i.e., method-of-moments (MoM) [45] for its numerical solution. In doing so, the foam domain is discretized into *n* cells with dimensions denoted as Δ, so that the dielectric constant and the total electric field are essentially constant over each cell. The unknown total electric field inside the domain can be represented using sub-domain pulse-basis function with unknown weight *w* as

(5) $$E\left(r\right)=\sum_{l=1}^{n}w_{l}E_{l}\left(r\right).$$

Here, the discretized electric field $E_{l}$ is defined as

(6) $$E_{l}\left(r\right)=\left\{\begin{array}{c} 1\;\;\forall (x,y)\in \mathrm{cell}\;l\\ \;0\;\;\;\mathrm{otherwise} \end{array}\right.$$

Then, Equation (2) is written as

(7) $$\begin{array}{c} \sum_{l=1}^{n}w_{l}E_{l}\left(r\right)=E_{\mathrm{inc}}\left(r\right)-\frac{jk^{2}}{4}\sum_{l=1}^{n}\left(\epsilon_{r_{l}}-1\right)w_{l}E_{l}\left(r\right)\underset{\Delta_{l}}{\;\iint \;}H_{0}^{2}\left(r,r^{\prime }\right)dr^{\prime }+\\ \;\frac{jk^{2}}{4}\sum_{l=1}^{n}\left(\epsilon_{r_{l}}-1\right)w_{l}E_{l}\left(r\right)\underset{\Delta_{l}}{\;\iint \;}H_{0}^{2}\left(r_{\mathrm{im}},r^{\prime }\right)dr^{\prime }, \end{array}$$

where $H_{0}^{2}$ is Hankel function of second kind and zero order. Further, after dot product, denoted by the operator $\langle \;\cdot \;\rangle$, of Equation (7) with the test function $E_{m}\left(r\right)$, we obtain

(8) $$\begin{array}{c} \sum_{l=1}^{n}w_{l}\langle E_{m}\left(r\right),E_{l}\left(r\right)\rangle =\langle E_{m}\left(r\right),E_{\mathrm{inc}}\left(r\right)\rangle -\frac{jk^{2}}{4}\sum_{l=1}^{n}\left(\epsilon_{r_{l}}-1\right)w_{l}\langle E_{m}\left(r\right),E_{l}\left(r\right)\rangle \underset{\Delta_{l}}{\;\iint \;}H_{0}^{2}\left(r,r^{\prime }\right)dr^{\prime }\\ \;+\frac{jk^{2}}{4}\sum_{l=1}^{n}\left(\epsilon_{r_{l}}-1\right)w_{l}\langle E_{m}\left(r\right),E_{l}\left(r\right)\rangle \underset{\Delta_{l}}{\;\iint \;}H_{0}^{2}\left(r_{\mathrm{im}},r^{\prime }\right)dr^{\prime }. \end{array}$$

Applying point collocation, i.e., choosing the test function as $E_{m}\left(r\right)=\delta_{m}\left(r\right)$ where δ is the delta function, we obtain the following matrix equation of the form

(9) $$\sum_{l=1}^{n}Z_{ml}w_{l}=E_{{\mathrm{inc}}_{m}}=\langle \delta_{m}\left(r\right),E_{\mathrm{inc}}\left(r\right)\rangle ,$$

where

$$\begin{array}{c} Z_{ml}=\langle \delta_{m}\left(r\right),E_{l}\left(r\right)\rangle +\frac{jk^{2}}{4}\left(\epsilon_{r_{l}}-1\right)\langle \delta_{m}\left(r\right),E_{l}\left(r\right)\rangle \underset{\Delta_{l}}{\;\iint \;}H_{0}^{2}\left(r,r^{\prime }\right)dr^{\prime }\\ \;-\frac{jk^{2}}{4}\left(\epsilon_{r_{l}}-1\right)\langle \delta_{m}\left(r\right),E_{l}\left(r\right)\rangle \underset{\Delta_{l}}{\;\iint \;}H_{0}^{2}\left(r_{\mathrm{im}},r^{\prime }\right)dr^{\prime }. \end{array}$$

The approximate solution of the surface integral in Equation (8), following [46] become

$$\frac{jk^{2}}{4}\underset{\Delta_{l}}{\;\iint \;}H_{0}^{2}\left(r,r^{\prime }\right)dr^{\prime }=\left\{\begin{array}{cc} \frac{j}{2}\left[\pi kaH_{1}^{2}\left(ka\right)-2j\right], & \forall \;m=l\\ \frac{j\pi ka}{2}J_{1}\left(ka\right)H_{0}^{2}\left(k\sqrt{{\left(x_{m}-x_{l}\right)}^{2}+{\left(y_{m}-y_{l}\right)}^{2}}\right), & \forall \;m\neq l \end{array}\right.$$

where $H_{1}^{2}$ is the Hankel function of second kind and first order, *a* is the radius of equivalent circular region having same area of the discretized cell, and $J_{1}$ is the Bessel function of first kind. To solve for the system of linear equations in Equation (9), generalized minimal residual method (GMRES) [47] is employed. Upon calculation of the unknown weights, the scattered electric field is evaluated at the transceiver points as

(10) $$\begin{array}{c} E_{\mathrm{sct}}\left(r\right)=-j\frac{\pi k}{2}\sum_{l=1}^{n}\left(\epsilon_{r_{l}}-1\right)w_{l}a_{l}J_{1}\left(ka_{l}\right)\left[H_{0}^{2}\left(k\sqrt{{\left(x-x_{l}\right)}^{2}+{\left(y-y_{l}\right)}^{2}}\right)-\right.\\ \left.H_{0}^{2}\left(k\sqrt{{\left(x_{\mathrm{im}}-x_{l}\right)}^{2}+{\left(y_{\mathrm{im}}-y_{l}\right)}^{2}}\right)\right]. \end{array}$$

Note that in Equation (7), the term $E_{\mathrm{inc}}$ contains both the transmitted signal and the reflected signal from the PEC in the absence of the foam.

### 2.2. Parametric Model for Moisture Distribution

The dielectric values used to represent moisture variations are generated numerically, based on the dielectric characterization of the polymer foam in laboratory environment. In the characterization, cavity perturbation method was used for dry sample and measurement at different moisture levels were performed with samples that cover all the cross section of a WR340 waveguide and using transmission reflection method [48]. The moisture content is calculated based on the *wet-basis*, i.e.,

(11) $$M=\frac{W_{m}-W_{d}}{W_{m}}\times 100,$$

where *M* is the moisture percentage, $W_{m}$ is the weight of the foam sample after adding the water, and $W_{d}$ is the weight of the dry sample. At the first step, we obtained the dielectric constant associated with the 0% moisture level. Then, a certain amount of water is added manually and the dielectric constant is recorded in each level. Thus, a relationship between the *wet-basis* moisture content $M_{\mathrm{meas}}$ and its corresponding real part and the imaginary part of the dielectric value is obtained and given as

(12) $$\theta ={\bar{a}}_{\theta }\exp\left({\bar{b}}_{\theta }M_{\mathrm{meas}}\right),$$

where $\theta =\{\epsilon_{r}^{\prime },\;\epsilon_{r}^{\prime \prime }\}$ denotes the material parameters. Numerical values for ${\bar{a}}_{\theta }$ and ${\bar{b}}_{\theta }$ are given in Table 1 where the error bounds for the fitted coefficients are defined by $\delta_{a_{\theta }}$ and $\delta_{b_{\theta }}$. Based on (12), the real part of relative dielectric constant vary in the range of 1.164–3.255 and imaginary part vary between 0.017–0.276 for *wet-basis* moisture content from 0% to 90%, respectively.

Further, we assume that the moisture field variation *M* in the foam is smooth. To generate such a random field, we utilise an anisotropic covariance structure *C* with its elements calculated as [49]

(13) $$C_{ij}=\exp\left\{-\frac{1}{2}\left(\frac{{∥x_{i}-x_{j}∥}^{2}}{l_{x}^{2}}+\frac{{∥y_{i}-y_{j}∥}^{2}}{l_{y}^{2}}\right)\right\}.$$

Here, $i,j=1,\ldots ,N_{n}$ and $l_{x}$, $l_{y}$ are the characteristic length components. $N_{n}$ denotes the number of pixels. In practice, the characteristic lengths affect the moisture distribution in x, and y directions. To generate simulated moisture samples, the uncertainties in the dielectric characterization is also considered, and hence Equation (12) is replaced by

(14) $$\theta =a_{\theta }\exp\left(b_{\theta }M\right),$$

where $a_{\theta },b_{\theta }$ are random variables such that $a_{\theta }∼U\left({\bar{a}}_{\theta }-\delta_{a_{\theta }},{\bar{a}}_{\theta }+\delta_{a_{\theta }}\right)$ and $b_{\theta }∼U({\bar{b}}_{\theta }-\delta_{b_{\theta }}$, ${\bar{b}}_{\theta }+\delta_{b_{\theta }}$), where U denotes the uniform distribution. Numerical values for $\delta_{a_{\theta }}$ and $\delta_{b_{\theta }}$ are given in Table 1. The moisture content distribution in each sample *M* can be expressed as

(15) $$M=M^{*}𝟙+\delta_{M}LZ,$$

where 𝟙 is an all-ones vector, *L* is the lower triangular matrix of the Cholesky factorization of the covariance *C*, *Z* is a standard normal random vector, $M^{*}$ and $\delta_{M}$ are the mean and standard deviation of the moisture content field, respectively. A pseudo-code for generating a sample is given below.

**Figure 3:**
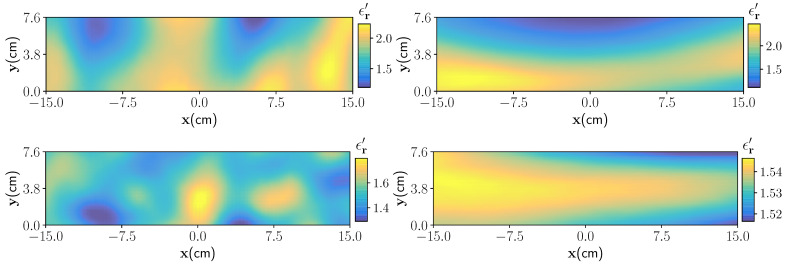
Four realisation of the moisture distribution with different correlation lengths, and mean and standard deviation parameters. In the top right figure the characteristic parameter are $l_{x}=3$ cm and $l_{y}=7$ cm, and for the top left figure characteristic parameter are $l_{x}=18$ cm and $l_{y}=4$ cm. Wet-spots ($l_{x}=3$ cm, and $l_{y}=3$ cm) and nearly homogeneous moisture distribution ($l_{x}=30$ cm, $l_{y}=7$ cm, and $\delta_{M}=2\%$) are shown in bottom left and right figures, respectively.

In Algorithm 1, the terms $x_{\min}=2$ cm, $x_{\max}=30$ cm, $y_{\min}=2$ cm, and $y_{\max}=7.6$ cm denote the chosen minimum and maximum dimensions in the x and y directions of the foam domain, respectively. U denotes the uniform distribution. Four randomized draws of moisture distribution are shown in Figure 3.
**Algorithm 1** Pseudocode for generating the moisture distribution. Note that a small diagonal component is added in matrix *C* to ensure the positive definiteness.1:$M^{*}∼U\left(0,50\right)\;\%$, $\delta_{M}∼U\left(2,20\right)\;\%$2:$c_{x}∼U\left(x_{\min},x_{\max}\right),\;c_{y}∼U\left(y_{\min},y_{\max}\right)$3:*C* = $\mathrm{AnisotropicCovariance}(c_{x},c_{y},x,y)$4:*L* = Cholesky(C)5:*M* = $M^{*}\;\mathrm{ones}\left(N_{n}\right)+\delta_{M}L\;\mathrm{randn}\left(N_{n}\right)$6:Calculate $\epsilon_{r}^{\prime }$, $\epsilon_{r}^{\prime \prime }$ using Equation (14)

## 3. Inverse Problem: Convolutional Neural Network

**Figure 4:**
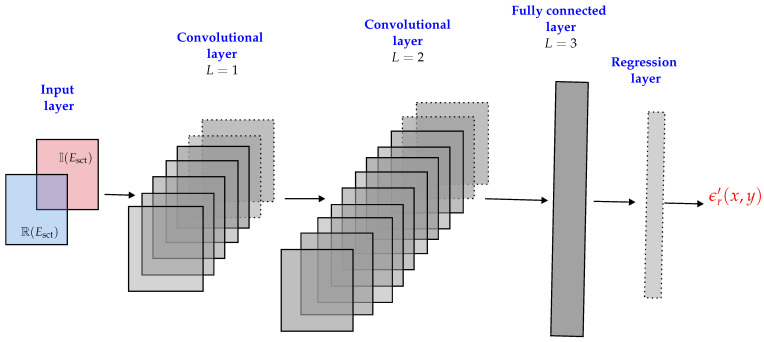
The architecture of the convolutional neural network used in this study.

In this study, a convolutional neural networks (CNN) is applied to estimate the moisture distribution of porous foam from scattered electric field data. The CNN $H_{w,b}\left(E_{\mathrm{sct}}\right)$ is trained to map from an input space $E_{\mathrm{sct}}\in R^{7\times 7\times 2}$ to Θ$\in R^{300\times 1}$ (vectorized moisture content distribution in terms of real part of the dielectric constant $\epsilon_{r}^{\prime }$). The network architecture used in this work comprises two convolution layers and two fully connected layers. The network architecture is shown pictorially in Figure 4. The input layer consists of two channels where the real part (channel 1) and imaginary part (channel 2) of the complex valued scattered electric data, i.e., $E_{\mathrm{sct}}$ are given as an input. The convolutional layers L=1 and L=2 have 20 and 30 channels with non-linear Rectified Linear Unit (ReLU) activation function and spatial filter of size 3×3 is chosen for both the layers. The fully connected layer L=3 has an output of size 340×1. As for the estimation of $\epsilon_{r}^{\prime }\left(x,y\right)$, an adequate resolution of the moisture distribution field of around x×y=1 cm×0.76 cm is chosen. Thus, the output layer has a size of 300×1. Note here that, moisture distribution is estimated in terms of real part of the dielectric constant only. Including the imaginary part of the dielectric constant is straightforward but it will increase the computational load.

The convolutional neural network (CNN) is trained using a dataset comprising of moisture content distribution $\left\{\Theta_{m}\right\}$ and corresponding scattered electric field parameters $\left\{E_{{\mathrm{sct}}_{m}}\right\}$, $m=1,\ldots ,N_{m}$. $N_{m}$ denotes the number of samples in the dataset. The generation of such a dataset is described below. In the training phase, the goal is to find biases *b* and weights *w* that minimize the discrepancy between $\left\{\Theta_{\ell }\right\}$ and the values estimated by the network $\left\{H_{w,b}\left(X_{\ell }\right)\right\}$. In this work, we minimize the quadratic loss function

(16) $$f\left(w,b;{\left\{E_{\mathrm{sct}}\right\}}_{m}\right)=\frac{1}{N_{m}}\sum_{m=1}^{N_{m}}{∥H_{w,b}\left(E_{\mathrm{sct}}\right)-\Theta_{m}∥}^{2},$$

to obtain the network parameters, biases, and weights of the network. For the network training process, the Adaptive moment estimation (Adam) optimizer [50] is chosen, with the batch size of 150 samples and epoch setting as 2000. The learning rates are set to $1\times 10^{-4}$ through out the training. All the computations were performed in a Python library TensorFlow [51] on a local computer with the configuration of 32 GB access memory, Intel Core(TM) i7-7820HQ central processing unit, and Nvidia Quadro M2200 graphic unit. The training of the network takes about 5 h.

### 3.1. Training, Validation, and Test Datasets

An initial dataset of $N_{m}$ = 10,000 samples containing complex scattered electric field response and corresponding moisture distribution is built. Here, the scattered electric field data is generated using 2-D MoM with pulse basis and point matching techniques at 8.3 GHz frequency and by discretizing the foam into 100×30 pixels. Note that the lower frequency point is chosen from X-band as it offers to simulate the 2-D full-wave electromagnetic simulations with less computational load and low degree of non-linearity [52,53]. The physical parameters $\epsilon_{r}^{\prime }$ and $\epsilon_{r}^{\prime \prime }$ for each sample were drawn using the framework discussed earlier. Furthermore, five copies of the dataset are created by adding noise between 1% to 3% to the scattering data. The noise is added (following [54]) to each response of the complex electric field of the dataset as

(17) $$E_{{\mathrm{sct}}_{\mathrm{noise}}}=E_{\mathrm{sct}}+\max\left(E_{\mathrm{sct}}\right)\frac{\beta }{\sqrt{2}}\left(\delta_{1}+j\delta_{2}\right),$$

where $\max(E_{\mathrm{sct}})$ is the maximum value of the scattered electric field, the coefficient $\delta_{1}∼U\left(-1,1\right)$ and $\delta_{2}∼U\left(-1,1\right)$ are two real vectors whose elements are sampled from uniform distribution. The term β denotes the noise levels and sampled as β∼U(0.01,0.03). Thus, leading to the total number of samples in the training dataset set to $N_{m}$ = 60,000 where the complex electric field values are vectorised in real and imaginary parts. In addition, 2000 samples are generated following the same procedures as a validation dataset. The noise is added to validation dataset similarly as for the training samples.

Furthermore, a test dataset with 1000 samples was generated using denser discretization in MoM computation. A different discretization was chosen to ignore “inverse crime”, i.e., the use of the same computational model or same grid settings to generate both training and test datasets. Otherwise, the same grid setting or the computational model may potentially lead to a situation where severe modelling errors are ignored and hence giving false impression on the accuracy of the estimates [55].

### 3.2. Reconstruction Results

This section gives results to evaluate the performance of the proposed neural network based estimation scheme. We applied the trained neural network to estimate the moisture field of the test datasets. The results are shown for four cases with low and moderate moisture levels, and high and nearly homogeneous moisture case.

#### 3.2.1. Sample with Low, and Moderate Moisture Content

**Figure 5:**
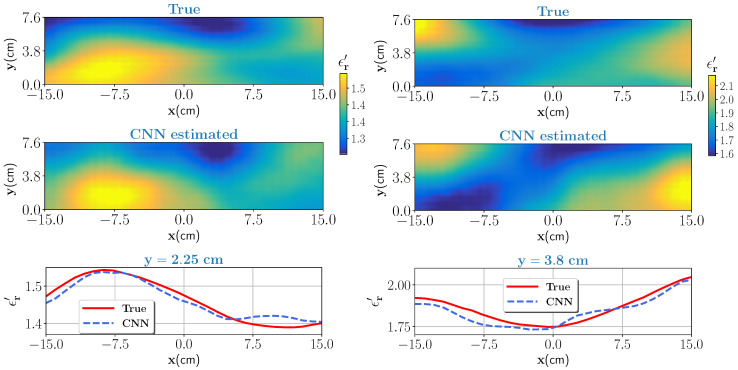
Low moisture case (left column): top figure shows the true profile and middle figure is the estimate from the CNN. Bottom figure compares the pixel values for the true and estimated profile at y=2.25 cm data line. Moderate moisture case (right column): same caption of the low moisture case except for the bottom figure where pixel values are compared for data line y=3.8 cm.

Two test samples with low (0–25%), and moderate (25–50%) wet-basis moisture contents are chosen as a first test case. As per the dielectric characterization, the real part of the dielectric constant value in the low moisture case varies approximately between 1.16 and 1.52 and for the moderate moisture case between 1.52 and 2.1. The corresponding scattered electric fields are measured and given as an input to the trained CNN. The noise level is set to β=0.03, see Equation (17). The true test samples and estimated outputs from the CNN for the low moisture and for the moderate moisture are shown in Figure 5 (left column) and Figure 5 (right column), respectively. Further, to assess the closeness of the estimates, pixel values on data line z=2.25 cm for low moisture case and pixel values on data line z=3.8 for moderate moisture are visualized and shown in the bottom of Figure 5. In both cases, the CNN estimated output closely matches the ground truth. Estimation accuracy is evaluated by comparing the profile similarity index, denoted here as κ, which is evaluated as

(18) $$\kappa =\frac{{\;\iint \;}_{\Omega_{\mathrm{foam}}}\bar{\epsilon_{r_{\mathrm{CNN}}}^{\prime }}\;\bar{\epsilon_{r_{\mathrm{True}}}^{\prime }}dxdy}{\sqrt{{\;\iint \;}_{\Omega_{\mathrm{foam}}}{\left(\bar{\epsilon_{r_{\mathrm{CNN}}}^{\prime }}\right)}^{2}dxdy}\sqrt{{\;\iint \;}_{\Omega_{\mathrm{foam}}}{\left(\bar{\epsilon_{r_{\mathrm{True}}}^{\prime }}\right)}^{2}dxdy}}.$$

The term $\bar{\epsilon_{r_{\mathrm{CNN}}}^{\prime }}=\epsilon_{r_{\mathrm{CNN}}}^{\prime }-\langle \epsilon_{r_{\mathrm{CNN}}}^{\prime }\rangle$, and $\bar{\epsilon_{r_{\mathrm{True}}}^{\prime }}=\epsilon_{r_{\mathrm{True}}}^{\prime }-\langle \epsilon_{r_{\mathrm{True}}}^{\prime }\rangle$. The operator $\langle \;\cdot \;\rangle$ is the mean operator. For the κ, its values vary between 0 and 1. As it gets closer to 1, the estimated profile is closer to the ground truth. The performance metrics values for low and moderate moisture cases are shown in Table 2.

For both cases, κ values indicate that estimated profiles are similar to the ground truth. Note that we interpolated the number of pixels in the true profile to correspond with the pixels in the estimated profile to calculate κ.

#### 3.2.2. Sample with High Moisture Distribution

**Figure 6:**
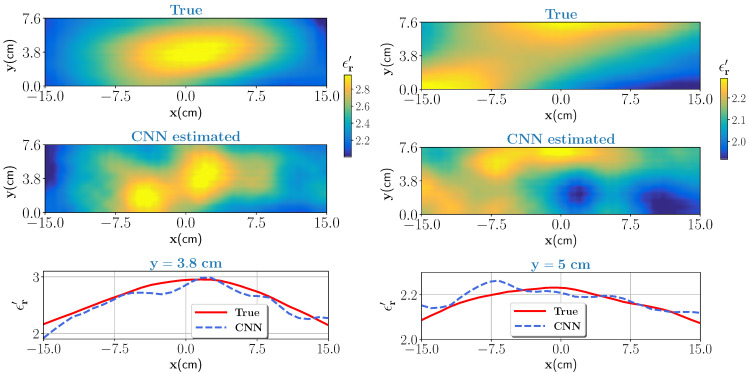
High moisture case with high variation (left column): top figure shows the true profile and middle figure is the estimate from the CNN. Bottom figure compares the pixel values for the true and estimated profile at y=3.8 cm data line. Nearly homogeneous case (right column): same caption except for the bottom figure where pixel values are compared for data line y=5 cm.

In the actual drying process, it is very likely that the moisture variation at the inlet has high moisture levels. Considering this scenario, two special cases of moisture distribution are considered. In the first case, we consider the moisture levels with variation between 50% to 70% with corresponding real part of dielectric constant between 2.1–2.95. For the second case, the moisture levels are high but minor variations in the moisture, between 52% to 55%, is assumed (nearly homogeneous). The corresponding scattered electric fields are measured and given as an input to the trained CNN. The noise level is set to β=0.03. The true test samples and estimated outputs from the CNN for the high moisture case with high variations and nearly homogeneous are shown in Figure 6 (left column) and Figure 6 (right column), respectively. Pixel values, as similar in the last section, are compared against the true case and shown in bottom for respective cases. For both cases, the estimated output is close to the ground truth. The profile similarity index, κ, values as shown in Table 3 indicate that estimated output is fairly close to the ground truth.

#### 3.2.3. Error Statistics

**Figure 7:**
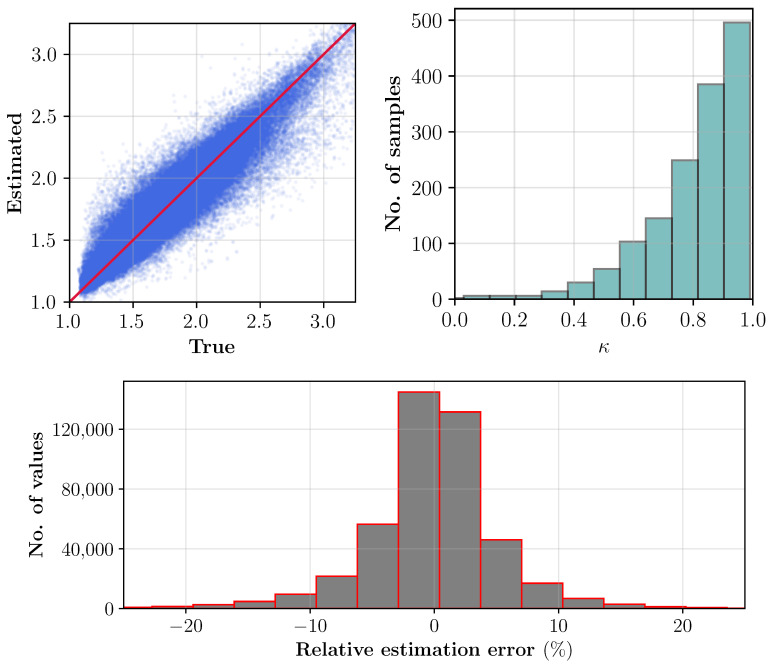
Top **left**: estimated pixel values of the $\epsilon_{r}^{\prime }\left(x,y\right)$ for the test dataset with 1000 samples. Top **right**: histogram of factor κ. **Bottom**: difference between the estimated and true values (relative estimation error) of the real part of the dielectric constant for the total number of test samples.

In the test dataset, for each sample the noise is added and its level is chosen from β∼U(0.01,0.03). Estimates of the $\epsilon_{r}^{\prime }$ for the whole test data are shown pixel-wise in Figure 7 (top left). Aside, the profile similarity index for each sample is compared against the respective true case and shown in Figure 7 (top right). The figure also includes a relative estimation error histogram. Uncertainties in the estimations can be seen mainly due to uncertainties in dielectric’s characterization (see Table 1) and higher noise levels. Specifically, with uncertainties in dielectric characterization, samples with same moisture levels are not unique in dielectric values. Nonetheless, the overall estimation success of the trained CNN on the test dataset for most samples are fairly good.

## 4. Experimental Setup and Result

**Figure 8:**
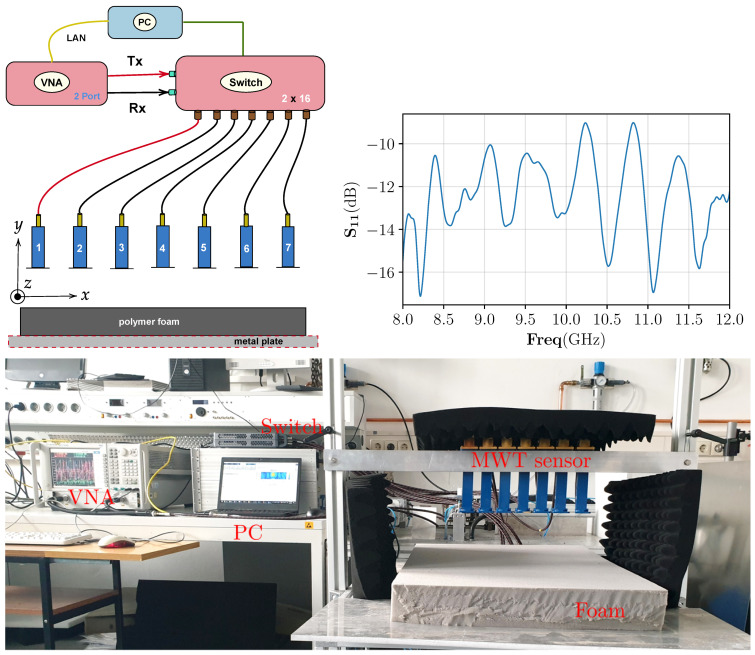
Top **left**: data acquisition scheme for the MWT measurement from the sensor array with X-band open waveguide antennas. Top **right**: the $S_{11}$ response of the WR-90 waveguide antenna. **Bottom**: prototype of MWT sensor array used in this study to generate measurement data. This system is developed at KIT, Germany and has been integrated with the HEPHASITOS technology.

In this section, the trained CNN performance is tested on the data from our developed MWT experimental prototype. The MWT experimental prototype consist of 7 WR90 open-ended waveguide antennas and placed over the foam of width = 50 cm, height = 7.6 cm, and length = 75 cm, respectively. The distance of the antenna to the top surface of the polymer foam is 5 cm, and the center to center distance between two adjacent antennas is 5 cm. Antennas are fixed and placed in free-space from −15 cm to 15 cm along the x-axis. For data acquisition, antennas are connected to the Agilent N5224A vector network analyzer (VNA) via a P9164C 2×16 USB Solid state switch matrix. Phase stable cables (with phase stability of $3^{\circ }$ at maximum frequency) are used for the connection. Communication between the VNA, switch, and the controlling computer is accomplished through the Ethernet cable. The block diagram of the data acquisition scheme and the $S_{11}$ (return-loss) response of a WR-90 waveguide antenna are shown in Figure 8 top left and top right, respectively. The data acquisition process and image reconstruction process (<1 s) is entirely automated using MATLAB. The measured scattered electric field data, in terms of scattering parameter, is acquired at 8.3 GHz frequency at cross-section of z=0 cm and takes around 20 s. Since the CNN network is trained on electric field data instead of scattering parameter, calibration scheme in [56] is employed for its conversion.

**Figure 9:**
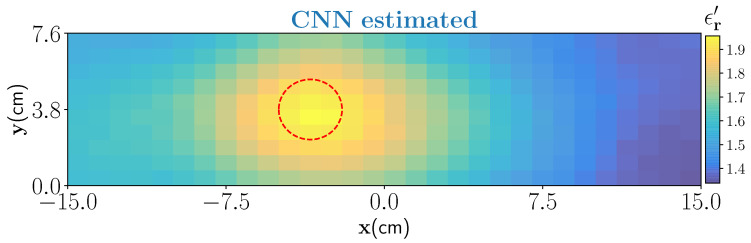
CNN estimation of cylindrical Teflon resin placed inside the foam. The true location of the target is marked by red-dash circle.

As a first example, we have considered a PTFE Teflon ($\epsilon_{r}^{\prime }\approx 2.1$) material with cylindrical shape (diameter of 2.25 cm) and placed inside the foam through an incision on the top surface. The reason for choosing this target is twofold. First, it will act as a benchmark target to test if the estimated dielectric values by the CNN are correct as the true value is well in the range of our interest. An approximate location of the target inside the foam is centered at (−4.5 cm,3.8 cm,0 cm). Second, to test the overall generalization capabilities of the trained architecture for identifying targets not seen as a ground truth while its training. The estimated output from the CNN is shown in Figure 9. Estimated result shows that the target is satisfactorily estimated by the network but it is slightly overestimated in the shape. The overestimation of the shape is predominately due to the smoothness model used in the training. However, note that the aim of our work is not accurate shape reconstruction and finding the locations of dominant of wet-spots is sufficient to design control strategies.

**Figure 10:**
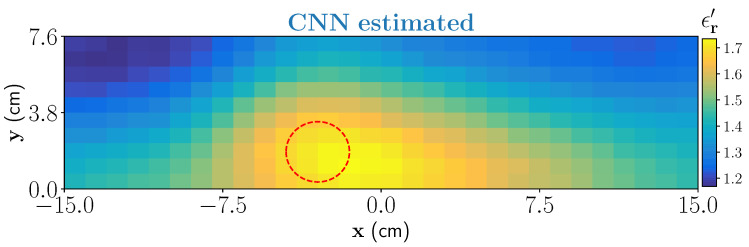
CNN estimation of one dominant wet-spot with 43% moisture inside the foam.

In the second example, we have considered a moisture wet-spot inside the foam. To create the wet-spot moisture target, a spherical foam of diameter 2.5 and with 43% wet-basis moisture level ($\epsilon_{r}\approx 1.81$−j0.079) is chosen. An approximate location of the target inside the foam is centered at (−3.25 cm,1.85 cm,0 cm). The estimated output from the CNN is shown in Figure 10. Estimated result shows that the network can satisfactorily locate the wet-spot which is placed around the bottom of the foam. The estimated real part of the dielectric constant corresponds between 37% and 39% of moisture level in the wet-spot.

## 5. Conclusions

In this paper, a neural network based method for the microwave tomography is developed for real-time moisture estimation in a polymer foam. The neural network is trained with synthetic data generated using two-dimensional scattering model based on method of moment formulation. Furthermore, in the scattering model, a parametric model based on the dielectric characterization of the foam is utilized to generate moisture samples. The network performance is tested with numerical data and experimental data from the constructed MWT prototype. Results shows the capability of the present method to be used in real-time moisture estimation. Here, the studied microwave imaging modality is applied to recover moisture content distribution inside a porous foam but the framework is applicable to investigate other material types together with different physical parameters. In the final stage, the estimated moisture information will be utilized in feed-forward loop of the intelligent control block of the industrial drying system.

It was observed in the experimental results that the real part of the dielectric constant in the estimation are slightly underestimated which are caused due to modeling errors, i.e., 3D measurements and 2D forward model and small uncertainties in the dielectric characterization of the foam, respectively. Henceforth, for our work the scope for improvement lies in the uncertainty quantification (UQ). In general, UQ in deep neural networks is a very active research topic and several approaches have been proposed and studied, see, e.g., recent reviews [57,58]. Furthermore, we also observe that in the estimates the background information, i.e., the dry part is not well distinguishable. This mainly due to the Gaussian based covariance structure used for generating moisture distribution. One solution is to use covariance structure models with a scaling factor such as Matérn class [49].

## Figures and Tables

**Table 1:** Material model parameters.

	${\bar{a}}_{\theta }$	$\delta_{a_{\theta }}$	${\bar{b}}_{\theta }$	$\delta_{b_{\theta }}$
$\epsilon_{r}^{\prime }$	1.085	0.01591	0.01256	0.00062
$\epsilon_{r}^{\prime \prime }$	0.03021	0.0025	0.02249	0.0009

**Table 2:** κ for low and moderate moisture case.

	Low Moisture	Moderate Moisture
κ	0.9558	0.9361

**Table 3:** κ for high moisture case.

	High Variation	Homogeneous
κ	0.923	0.883

## Data Availability

The data presented in this study are available on request from the corresponding authors.
